# Pollution Risk Assessment of Potentially Toxic Elements in Soils Using Characterization and Microbiological Analysis: The Case of a Rare and Precious Metal Mining Site in Wuzhou, Guangxi

**DOI:** 10.3390/toxics13040270

**Published:** 2025-04-02

**Authors:** Yi Sun, Zixuan Yang, Kun Dong, Fujiang Hui, Dunqiu Wang, Yecheng Huang

**Affiliations:** 1 Hubei Key Laboratory of Biologic Resources Protection and Utilization, Hubei Minzu University, Enshi 445000, China; 2 College of Forestry and Horticulture, Hubei Minzu University, Enshi 445000, China; 3 Guangxi Key Laboratory of Environmental Pollution Control Theory and Technology, Guilin University of Technology, Guilin 541004, China; 15293163375@163.com (Z.Y.); 2020005@glut.edu.cn (K.D.); h19507737192@139.com (F.H.); wangdunqiu@glut.edu.cn (D.W.); m18697920741@163.com (Y.H.)

**Keywords:** PTEs, soil pollution, characterization, risk assessment

## Abstract

To understand the characteristics of the pollution risk of potentially toxic elements (PTEs) at a rare and precious metal mining site in Guangxi and to provide scientific evidence for the comprehensive evaluation and soil remediation of PTE pollution at the site, the Cd, As, Co, Cu, Cr, Ni, Pb, and Zn contents of five areas were determined. Laboratory testing was conducted on five soil plots in the selected five suspected contaminated areas (electroplating workshop, sewage treatment area, and boiler room). Correlation analysis, infrared spectroscopy (FTIR), X-ray diffraction (XRD), and X-ray photoelectron spectroscopy (XPS) were used to evaluate and analyze PTE pollution. The average contents of Cd, Co, As, Pb, Zn, and Cu at the site were higher than the background values in the Guangxi soil. The Probability Mass Function (PMF) model was used to perform a source apportionment of the PTEs and determine the main pollution sources and their contribution rates. The results of the single factor pollution of the PTEs showed that Cd, Ar, and Cr were heavy pollutants, and Co was a light pollutant. The Nemerow comprehensive pollution index analysis showed that the study area was heavily polluted. The Earth accumulation index results show that Cd exhibited a very serious accumulation, Cu and Zn exhibited mild to moderate accumulations, and As and Co exhibited moderate accumulations. The FTIR results showed that C=O in the soil was chelated with PTEs in some samples, which weakened the characteristic peaks of C=O in proteins and polypeptides. The XRD results showed that cadmium hydroxide, lead oxide, and zinc hydroxide were present in the soil samples. The XPS results showed that the production of O^2−^ in the O 1s high-resolution spectra mainly came from the metal oxides produced by the polluting metals. Meanwhile, the microbial results showed that the pollution risk of PTEs affected the soil microbial community structure and diversity to some extent.

## 1. Introduction

Soil is the base of agricultural production [1] and provides a living environment that promotes the growth and reproduction of plants and microorganisms [2]. Potentially toxic elements (PTEs) in agricultural soils, particularly cadmium (Cd) and lead (Pb), pose significant threats to human health and the environment [3,4,5]. They originate from various human activities. The topsoil near agricultural soils with PTEs acts as a direct sink, promoting the transfer of PTEs into the food chain. Even at ambient levels, PTEs migrate under acid conditions. Because they cannot be degraded, they may cause secondary pollution in water bodies and exacerbate ecological damage [6,7,8,9]. Under natural conditions, the transportation of weathered rock mineral products by rivers is the main source of PTEs in sediments. With societal progress in urban development, agricultural and industrial activities in lake basins, including urban transportation, fossil fuel combustion, mining, and metal smelting, and the use of fertilizers and pesticides, soil environmental pollution has expanded [10].

PTEs in soil solutions tend to accumulate on soil surfaces by adsorption, and some of them might be bacterially transformed into methyl compounds that are more toxic for us [11,12,13]. Studying soil pollution by PTEs and effectively controlling it, minimizing the pollution caused to the soil by the source, can improve the ecology of a soil and ensure the health of people. Research on soil PTE pollution mainly involves studying the basic physical and chemical properties of soil, analyzing the characteristics of PTE content, particularly different forms and distributions of PTEs in time and space, and combining different pollution assessment methods (for example, the Nemerow index method, single pollution index method, ratio of secondary phase and primary phase method, and risk assessment coding method) to analyze pollution level and ecological risk [14,15]. Several such studies have been published [14,15,16,17].

In the rare and precious metal mining area of Guangxi, there are metallurgical facilities where indium is extracted from zinc oxide through wet smelting and the resulting zinc sulfate solution is used to produce lithopone and solid zinc sulfate. The industrial solid waste produced is sent to the rotary kiln production line to recover valuable metals, and the resulting product, zinc oxide, is used as a supplementary raw material for the wet extraction of the indium production line. In this area, there is also a significant presence of solid waste (abandoned structures, construction waste, and residue waste) that could act as a secondary source of environmental pollution. In hot spots, such as the rare and precious metal mining area in Guangxi, where soil pollution levels exceed soil pollution risk control standards, relevant parties should conduct soil risk assessments for PTEs. These soil pollution risk assessment reports must aim to provide a reference for human health risk prevention and scientific basis for their risk control measures and restoration efforts in accordance with national regulations and requirements.

The difference between this area and others is that previous research has shown that for plots with soil pollution levels exceeding the soil pollution risk control standards, relevant parties should conduct soil pollution risk assessments and take corresponding risk control measures in accordance with national regulations and the requirements of soil pollution risk assessment reports. The present study considered the soils of a rare and precious metal site in Guangxi Province as the research object. By measuring the contents of Cd, As, Cu, Cr, Ni, Pb, and Zn in the soil at the site, the single pollution and Nemerow indices were calculated. Infrared spectroscopy (FTIR), X-ray diffraction (XRD), and X-ray photoelectron spectroscopy (XPS) were used to evaluate the soil pollution status and potential ecological risks. Correlation analysis was conducted to provide a reference for soil pollution prevention and risk control at the site. The single factor pollution method, Nemerow index analysis method, and geoaccumulation index method were used to evaluate the potential toxic element pollution source characteristics, distribution, pollutant types, and concentrations of the site’s soils and conduct a risk assessment of soil pollution. The research results provide a reference for the treatment of contaminated soils in rare and precious metal mining areas.

## 2. Materials and Methods

### 2.1. Site Characteristics

**Figure 1:**
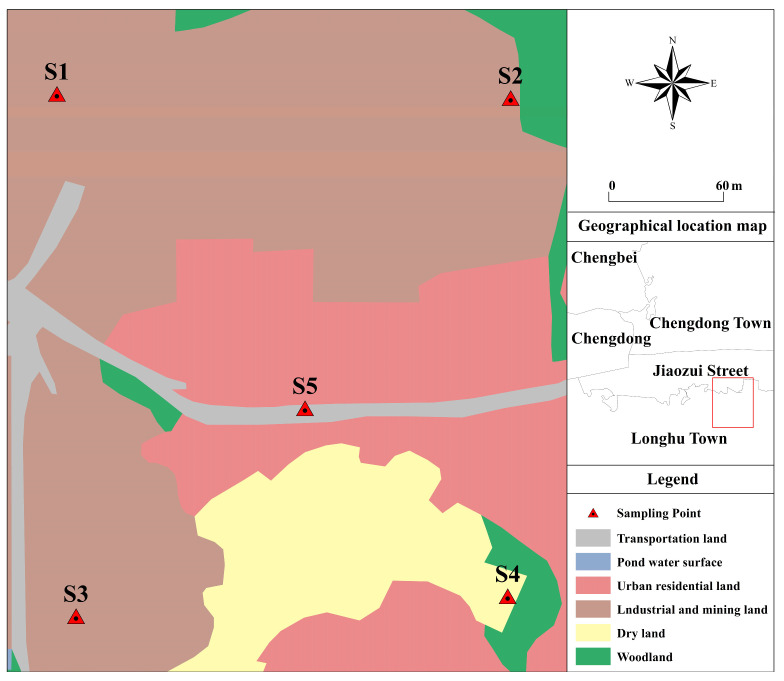
Overview of the study area. (S1–S5 are five sampling points).

The study area is a rare and precious metal site in Guangxi (Figure 1). It belongs to a subtropical monsoon climate with high temperatures (annual average temperature 21.6 °C) and is rainy and humid throughout the year. The site is generally high in the south and low in the north. Most surrounding areas contain industrial and residential buildings. The terrain of the area is relatively flat and there are no drinking water source protection areas, nature reserves, scenic spots, cultural relics, historic sites, or other environmentally sensitive objects that require special protection within 1 km of the site. The site primarily uses hydrometallurgy to produce secondary zinc oxide and refined iron ore from metallic zinc and iron slags.

This is a former mining site with metallurgical facilities. Excavation work is stopped, and the metallurgical production line is not in use. It awaits land restoration. Currently, the buildings and equipment in the plant area are dismantled, and the products and raw and auxiliary materials have been cleared and removed. The soil pollution status of the plots was investigated, and the area will be planned as a protective green space, storage, and logistics land, which will belong to the first class of land specified in the Standard for Soil Pollution Risk Management and Control of Construction Land for Soil Environmental Quality (GB36600-2018) [18].

The main process flow at this site was the extraction of indium from indium-containing zinc oxide via hydrometallurgy. The zinc sulfate solution produced was simultaneously used to produce lithopones and solid zinc sulfate. The industrial solid waste and metal waste produced in the process of hydrometallurgical indium extraction and lithopone production enter a rotary kiln production line to recover valuable metals, and the product, zinc oxide, was used as a supplementary raw material for hydrometallurgical indium extraction production. The production facilities in the plant area included a steam supply section, leaching slag yard, zinc barium white section, and steelmaking section.

### 2.2. Sample Collection and Processing

Based on preliminary and detailed investigations, monitoring points were planned to be set up in areas within the site that may be heavily polluted. Due to the use of a large amount of strong acidic materials in the production process, the tanks, valves, and other items of machinery are severely corroded, and the conveying pipelines and anti-corrosion lining are aging. Owing to leakage and seepage during the original production process, pollutants may have spread throughout the site, posing a significant threat to the safety of the soil, the groundwater, and the surrounding natural water environment. Due to years of outdated production activities, the soil at the site is polluted to varying degrees. The commonly used point sampling method is the five-point sampling method. First, the midpoint of the diagonal (in a square or rectangular sampling area, the diagonal refers to the straight line connecting the two diagonal vertices of the sampling area) was determined as the center sampling point, and then four points on the diagonal equidistant from the center sampling point were selected as sampling points. For S1–S5, we followed the five-point sampling method, where two samples of the same sample at each point are analyzed synchronously under identical conditions using the three-point sampling method. The soils at the rare and precious metal site were sampled from five plots, each with an area of 300 m^2^, representing soils with different concentrations of PTEs.

We conducted sampling in the survey area using the five-point sampling method in May 2024. And three replicate samples were measured at each sampling point. The points in the production area, sewage treatment area, slag yard, storage area, and rotary kiln exhaust outlet of the factory were labeled S1–S5. A five-point sampling method was adopted for the soil sample collection from the quadrats, and three parallel samples were selected from each quadrat. The soil samples were placed in polyethylene bags, and debris (rocks, leaves, garbage, etc.) was removed, followed by air drying, grinding, and sieving through a 160 mesh. The total amount of PTEs in the soil was extracted using nitric acid, hydrochloric acid, hydrofluoric acid, and a microwave digestion system. The soil samples (0.2 g) were weighed and sieved into a digestion tube. Five regions with relatively high concentrations of PTEs were selected for characterization. Microwave digestion was carried out according to a “6 nitric acid, 6 hydrochloric acid, 3 hydrofluoric acid” acid mixture. A total of 2 mL of nitric acid and 1 mL of hydrofluoric acid were added to the digested samples and placed on an electric heating plate to reduce the acid volume to about 1–2 mL, and the mixture was then cooled to room temperature, made to a fixed volume in a 25 mL colorimetric tube, and filtered with a 0.45 μm water system filter membrane. Three blank samples and three soil reference materials (gss-8) were run through the same process with each sample digestion batch. An inductively coupled plasma mass spectrometer (Perkin Elmer, icp8000, Waltham, MA, USA) was used to detect the Cd, Co, As, Pb, Zn, and Cu in the digested samples. The recovery rate of the certified reference materials was 91–97%, and the test method was accurate and reliable. The functional groups of the samples were analyzed using a Fourier-transform infrared spectrometer (Bruker, ten-sor27, Billerica, MA, USA). The test conditions were as follows: scanning range was 400~4000 cm^−1^, the scanning time was 16° min^−1^, and the resolution was 4 cm^−1^. An XRD diffractometer (Malvern Panalytical, X’Pert^3^, Almelo, The Netherlands) was used to scan the crystal structure of the samples. The scanning angle was 2 θ = 10°~90°, and the scanning speed was 5 (°) min^−1^. The obtained XRD spectra were analyzed using Jade software 9.0. X-ray photoelectron spectroscopy (Thermo Fisher, escalab 250xi, Waltham, MA, USA) was used to analyze the elemental composition of the material surface, and the tested elements were charge-corrected with C 1s = 284.8 EV binding energy. The pH of the soil samples was determined using a 2.5:1 water–soil ratio (V:m) extraction potentiometric method.

### 2.3. Impact Assessment Method of PTE Pollution in Soil

In this study, the single pollution, Nemerow, geoaccumulation, and potential ecological risk indices were used to evaluate PTE pollution at the site. The background values of the Cd, As, Cr, Ni, Pb, Zn, and Cu in Guangxi were 0.267 mg/kg, 20.80 mg/kg, 82.10 mg/kg, 26.60 mg/kg, 24.00 mg/kg, 75.60 mg/kg, and 27.80 mg/kg, respectively [19].

#### 2.3.1. Single Pollution Index Method

The single pollution index method evaluates the worst-performing indicator by comparing the measured data of each factor against the project’s environmental quality standards, thereby determining their pollution category [20,21]. The single pollution index method can strengthen the objectivity of index weight determination. The single factor index method was used for the evaluation. The pollution index was calculated by comparing the measured values of the soil samples with their threshold limits. This study used the screening values from the GB36600-2018 standard. Since this standard does not specify a limit value for zinc, the soil pollution risk screening guideline value for construction land (500 mg/kg) was adopted as its reference to assess its contamination level.

The formula used was the following:

(1) $$P_{i}=\frac{C_{i}}{S_{i}}$$

where *P_i_* is the pollution index of each *i*th PTE pollutant, *C_i_* is the concentration of each *i*th PTE pollutant, and *S_i_* represents the evaluation criterion for the *i*th PTE pollutant.

#### 2.3.2. Nemerow Index Method

The Nemerow index, which is a weighted multi-factor environmental quality index that considers both extreme values and prominent maximum values [22,23,24], highlights the evaluation factors with serious pollution. The classifications of the index are shown in Table 1.

The formula used was the following:

(2) $$P_{Comprehensive}=\sqrt{\frac{{P_{i}}^{2}+P_{max}^{2}}{2}}$$

where $P_{Comprehensive}$ represents the comprehensive pollution index of PTEs at the sampling point and $P_{max}$ represents the maximum value of the single factor pollution index of each PTE pollutant at the sampling point.

#### 2.3.3. Index of Geoaccumulation (I*geo*) Method

The index of geoaccumulation (I*geo*) method [25,26] for heavy metals is primarily used to study changes in the background value of PTE pollution caused by natural processes and the comprehensive impact of human activities on soil PTE pollution. The formula used was the following:

(3) $$I_{geo}={log}_{2}\left(\frac{C_{i}}{LB_{i}}\right)$$

where $I_{geo}$ represents the pollution index of geoaccumulation for PTEs, $C_{i}$ represents the measured concentration of PTEs, $B_{i}$ represents the background value of the PTEs used in this study (the soil background value of Guangxi Province was used in this study), and *L* is a constant. This is a coefficient that considers possible changes in background values caused by differences in rocks in different regions, and the value of *L* was 1.5 in this study. Classification of the index of geoaccumulation was shown in Table 2.

#### 2.3.4. PMF Model Construction

The PMF model was used to apportion sources to the PTE data and determine the main pollution sources. The PMF model is a method first proposed by Paatero et al. in 1994 [27] and uses the weight coefficients of variables to determine the error of pollution components. It iteratively calculates the main pollution sources and their rates of contribution using the least-squares method. Owing to its advantages, including not requiring detailed source component spectra, being able to decompose factor loadings, and utilizing data uncertainty for optimization, the PMF model is widely used in apportioning heavy metal sources in soils. The basic principle of the PMF model is to decompose the original matrix *X* (*n* × *m*) into a factor contribution matrix *G* (*n* × *p*), factor composition matrix *F* (*p* × *m*), and residual matrix *E* (*n* × *m*).

When the concentration of the chemical components was less than or equal to the corresponding method detection limit (*MDL*), the uncertainty (*U*) was calculated using Equation (4):

(4) $$U_{nc}=5/6\times MDL\;\;\;\;\;$$

When the concentration of the chemical components exceeded the corresponding *MDL*, *U* was calculated according to Equation (5):

(5) $$U_{nc}=\sqrt{{\left(c_{onc}\times EF\right)}^{2}+{\left(0.05\times MDL\right)}^{2}}$$

where *U_nc_* represents the uncertainty of the analyzed components (mg/kg); *MDL* represents the detection limit of the components (mg/kg); *C_onc_* represents the mass concentration of the components (mg/kg); and *EF* (error fraction) represents the error coefficient of each component.

The indicator judgments Q (robust) and Q (true) were both 2.63102, with an error of less than 10%, and the model was interpretable. The BS inspection rate exceeded 80%; therefore, the results were usable. (Weak represents the component with high measurement uncertainty in Table 3).

#### 2.3.5. Extraction and High-Throughput Sequencing of Microbial DNA

The soil samples were pretreated, and the DNA was extracted using a DNA Extraction Kit (E.Z.A^TM^mag-bindsoildnakit, Omega Bio-tek, Norcross, GA, USA). The target gene of the V3-V4 region was amplified by the 16S rRNA universal primers 341F (5′-CCTACGGGNGGCWGCAG-3′) and 805R (5′-GACTACHVGGGTATCTAATCC-3′). Illumina bridge PCR-compatible primers were used for the second round of amplification. The original data were spliced from MiSeq sequencing to distinguish the samples. Subsequently, the quality of the sequence was controlled and filtered, and the operational taxonomic unit (OTU) was used for the clustering and taxonomic analyses of species.

#### 2.3.6. Data Analysis and Processing

Excel 2016 was used to statistically analyze the content of PTEs in the soil, and SPSS 26 and R studio 4.3.0 were used to analyze the correlation between the PTEs in the soil.

## 3. Results

### 3.1. PTE Content in Soil Samples

The basic physical and chemical properties of the soil samples from the five sites were determined. The soil type in this area belongs to the following solid waste pollution type: soil pollution caused by the stacking or disposal of industrial and mining waste, sludge, and urban garbage on the surface. The pH of the samples was between 6.81 and 7.33. The pH values of the five points S1–S5 were 6.81, 7.10, 6.89, 7.21, and 7.33, respectively. The PTE contents varied significantly. The average contents of Cd, Co, As, Pb, Zn, and Cu were higher than the background values for the soil in Guangxi (Table 4). The coefficient of variation (CV) was used to characterize the spatial dispersion of the soil samples. Compared to the screening value of the standard for the control of the soil pollution risk of construction land for soil environmental quality (GB36600-2018), the sample points exceeding the standard values of Cd, Co, As, Cr, and Pb were 80%, 60%, 60%, 40%, and 20%, respectively. The CVs of Cd, Co, As, Cr, Ni, Pb, Zn, and Cu were 7–142%, of which the coefficients of variation of Cd, As, Pb, and Cu were all greater than 1, namely 1.03, 1.42, 1.04, and 1.24, respectively, indicating that the elemental contents of the different samples in this study were quite different, with a high degree of dispersion, and were greatly affected by external factors. No points exceeded the standards for soil Ni, Zn, and Cu, that is, the content was lower than the standard for controlling the soil pollution risks of construction land, and the average content of Cd, Co, and As in the soil exceeded the screening value. Compared to the background values of the soil in Guangxi, the average contents of Cd, Co, As, Pb, Zn, and Cu were higher. From these results, the scope and degree to which the standard was exceeded were relatively large, which may be due to the fact that raw materials, including metal waste residue and other raw and auxiliary materials, entered the ground during production, processing, storage, transportation, and pollution treatment due to rainwater scouring and leakage through surface cracks, resulting in a degree of pollution in the soil.

### 3.2. Assessment of PTE Pollution in Soil

#### 3.2.1. Single Factor and Nemerow Index Methods

The pollution intensities of the PTEs ranged from strong to weak as follows: Cd (7.154) > As (5.312) > Co (2.378) > Pb (0.660) > Cr (0.625) > Zn (0.375) > Ni (0.166) > Cu (0.038). Ni, Pb, Zn, and Cu belonged to the non-pollution level (Table 5). The average content of Cd ranged from 0.279 to 20.284 mg/kg, which was much higher than the background value of the soil. As was between 0.0735 and 21.260 mg/kg, which is classified as severe pollution, while Co was between 0.618 and 4.484 mg/kg, which is classified as mild pollution, but its average content was more than two times higher than the background value of the soil.

#### 3.2.2. Index of Geoaccumulation (I*geo*)

The average accumulation of PTEs in the soil of the five sites was Cd (8.481) > Pb (2.876) >As (1.789) > Co (1.608) > Cu (0.895) > Zn (0.729) > Ni (−0.675) > Cr (−1.526) (Table 6). Among these, the average value of the index of geoaccumulation of Cu and Zn was 0 ≤ I*geo* < 1, reflecting mild to moderate accumulation, and the average value of the index of geoaccumulation of Ni and Cr was less than 0, indicating an absence of accumulation.

Sample points with extremely serious cumulative pollution levels of Cd and moderate to strong cumulative pollution levels of Pb in the soil were distributed in the replacement section, slag yard area, and extraction and reverse extraction workshop. Pollution sources were mainly related to the migration and transformation of geological background elements caused by high background values of Cd and Pb in the soil, as well as the artificial transportation of metal waste and other raw and auxiliary materials. This result is consistent with the conclusion of previous studies that the main pollutants in the soil near smelting activities are As, Cd, and Pb [28,29].

### 3.3. Correlation Analysis and Source Apportionment of PTEs in Soil

**Figure 2:**
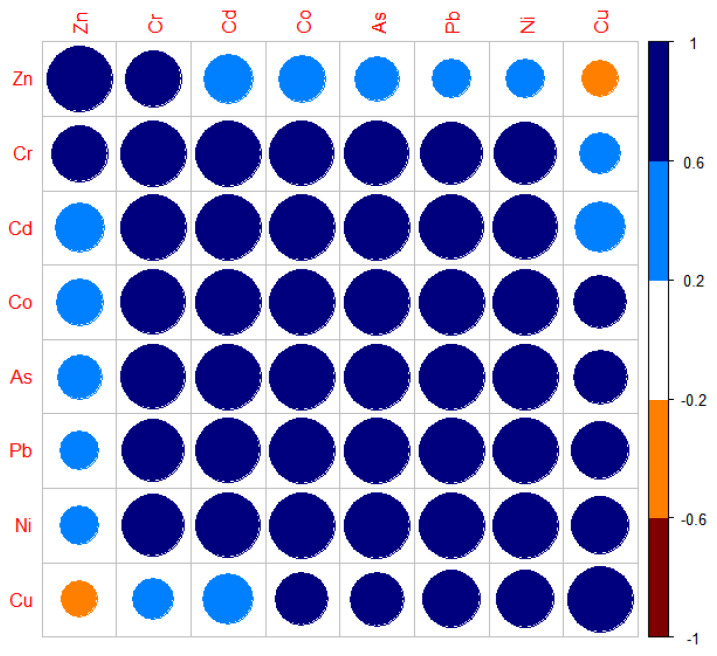
Correlation diagram of PTEs in soil.

SPSS 26 correlation analysis of the PTEs in the soil of sites showed that a *p* value < 0.01 signified an extremely significant correlation, 0.01 < *p* < 0.05 was a significant correlation, and *p* > 0.05 was not a correlation. Table 7 shows the correlation results of the PTEs in the soil at the five sites. Pb exhibited a very significant correlation with Ni, As, Co, and Cd, and a significant correlation with Cr. Ni exhibited a close relationship with Cr, As, Co, and Cd, showing a significant correlation. The main source of Ni in the soil was its geological form, and the pollution of Pb, As, and Co also reflected an anthropogenic impact caused by the construction waste and waste residue generated by the artificial metal waste residue and other raw and auxiliary materials in the production and leaching process areas. The pollution of soil Cd may have been caused by the discharge of industrial “three wastes” and the migration of toxic and harmful substances into the soil caused by a large number of metal warehouses. These correlations are consistent with the pollution of the soil at the site (see Figure 2). 

**Figure 3:**
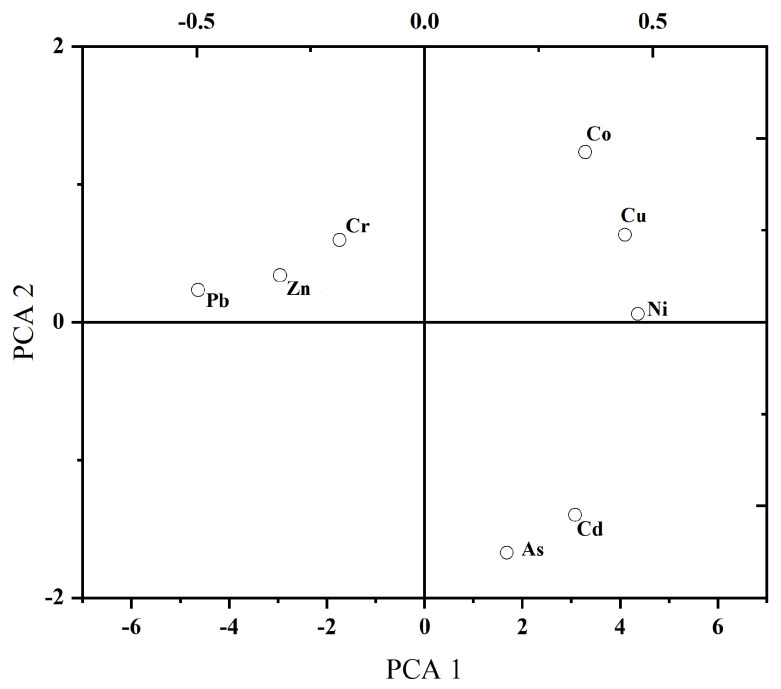
Soil PTE principal component analysis rotation space component diagram.

To further analyze the pollution sources of the PTEs, a principal component analysis of eight types of PTEs was carried out. Three principal components were extracted, and the characteristic root values exceeded unity. Principal component 1 contributed 39.86%, principal component 2 contributed 29.15%, and principal component 3 contributed 22.23%, and their cumulative contribution was 91.24% of the total variation. The results of the principal components of the PTEs in the soil are shown in Table 8. As shown in Table 8 and Figure 3, the loads of Cu, Ni, and Pb in principal component 1 were relatively high. According to the distribution of pollution characteristics, these four PTEs were distributed near production and leaching process sites, replacement sections, and slag yard areas, and thus may be caused by the presence of solid wastes such as abandoned structures, construction waste, and slag in the plot and smoke, wastewater, and slag generated in the process of the preparation of raw materials and smelting, as well as by emissions, dripping, and leakage in the production process. The average content of the Cd, Co, and Cu PTEs exceeded the soil background value in Guangxi, and the range of their CVs was 0.66–1.24, indicating that these elements may be affected by human activities and that their non-uniformity may also be caused by hydrothermal and invasive directional processes. Their morphological changes were caused by changes in the solubility of each mineral. Although Co showed a relatively high loading in principal component 2, its depth concentration remained below the standard threshold, which may be due to the additional sources of Co possibly due to the inclusion of some production waste residue in the backfill or the soil outside the site that entered the plot to cause pollution.

### 3.4. Source Apportionment of Potential Toxic Elements in PMF Model

**Figure 4:**
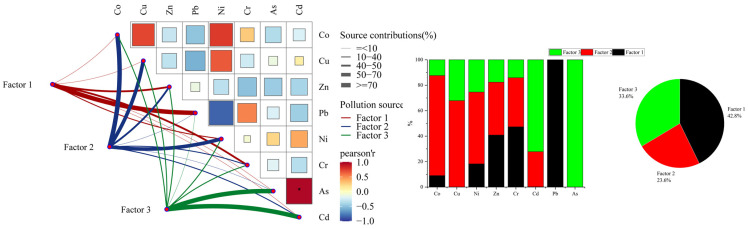
Factor contribution rate of PMF source resolution.

The PMF model was used to identify and distinguish the potential sources of toxic elements in the soil. Table 3 lists the optimal number of factors. In this study, the number of input factors was set to three, and the indicator judgments Q (Robust) and Q (True) were both 2.63102, with an error of less than 10%. This model was interpretable, and the BS inspection rate exceeded 80%; therefore, the results are usable. Factor contribution rate of PMF source resolution was shown in Figure 4.

The contribution of Pb to factor 1 was as high as 99.9%, followed by Cr with a contribution of 47.3% and Zn with a contribution of 40.9%. The correlation between Pb and Cr was positive and equaled 0.884, whereas the contributions of Cr and Zn were negative and equaled 0.726. The high contribution of Pb reflects the presence of different forms of Pb at this site. Pb mainly comes from the combustion of fossil fuels, and due to human activities (such as the use of refined zinc ore to produce zinc oxide) and the input of agricultural chemicals, Pb accumulates in the soil. The study area has a history of using coal as a fuel during winter. Because of the stable air in winter, a temperature gradient is formed and the cold air at the bottom cannot rise to carry away pollutants, leading to their accumulation in the lower atmosphere and subsequent deposition onto the soil surface. The complex correlation of Pb-Cr-Zn reveals that this precious metal site is affected by numerous pollutants derived from traffic dust and metal-processing waste, whereas the negative correlation of Cr-Zn suggests the possibility of cross-contamination from different processes. Factor 1 was defined as a combination of transportation and industrial sources.

The contribution of Co to factor 2 was 78.6%, while the contributions of Cu, Ni, Zn, and Cr were 67.9%, 56.4%, 41.6%, and 38.6%, respectively. The correlations between Co and Cu and Co and Ni were positive, equaling 0.7 and 0.76, respectively. Consistent with the characteristics of nonferrous metal smelting sources, the Co–Ni–Cu combination is typical of the alloy manufacturing and electronics industries. This may have originated from the dismantling of waste electronic products, metal melting, and the process of industrial solid waste produced during the wet process of indium extraction and lithopone production entering the rotary kiln production line to recover valuable metals. Therefore, factor 2 reflects the influence of metal smelting and electronics manufacturing.

Factor 3 showed absolute dominance and a strong positive correlation (0.96) between As (100%) and Cd (72%), indicating agricultural non-point source pollution. The high coupling of As-Cd is consistent with the long-term application of arsenic-containing pesticides and phosphorus fertilizers. There was a certain amount of farmland around the plot, and because of the legacy of heavy metal raw materials, especially the arsenic and cadmium elements associated with phosphorus fertilizers, they accumulated in the surface soil through agricultural cultivation. Thus, factor 3 also reflects agricultural sources. The mixed sources of transportation and industry contributed 42.8%, indicating that emissions from transportation and various industrial production processes around precious metal companies are important factors leading to the heavy metal pollution of soils. The metal smelting and electronics manufacturing industries accounted for 23.6%. Owing to the characteristics of their precious metal companies’ wet zinc-smelting production processes, these two industries often generate large amounts of heavy metal waste and harmful substances. Waste easily infiltrates the surrounding environment and becomes an important source of heavy metal pollution in the soil. Agricultural activities account for 33.6% of the total. Pesticides and fertilizers used in agricultural production, as well as manure produced from livestock and poultry farming, may contain heavy metals. The long-term and extensive use of these chemicals leads to their gradual accumulation in soil.

### 3.5. Characterization of Soil

#### 3.5.1. FTIR Analysis

**Figure 5:**
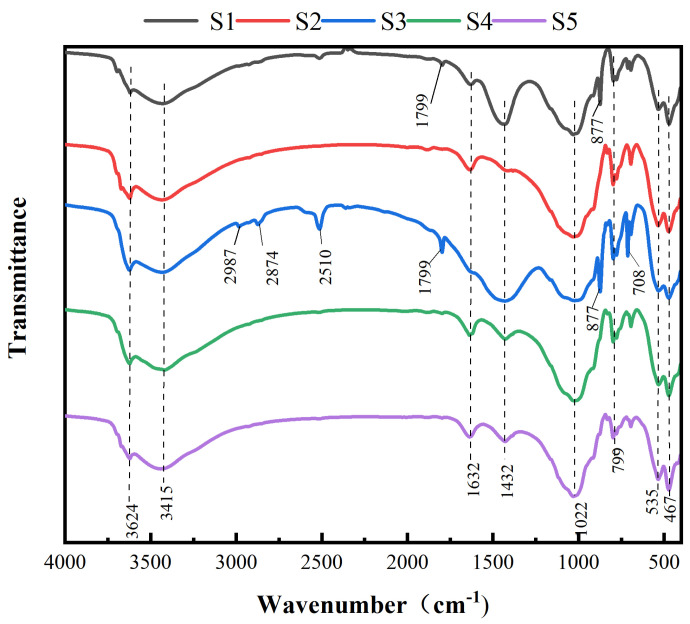
FTIR diagram of soil at five sample sites.

Figure 5 shows the infrared spectra of the soil at five different sampling sites. In the infrared spectra of these samples, there are characteristic peaks with a wavenumber of 3624 cm^−1^, which are mainly caused by the stretching vibration of N-H in the amino acids and nucleic acids in the soil. The wide peak at a wave number of 3415 cm^−1^ belongs to the associated hydroxyl -OH stretching vibration peak in carbohydrates, hemicellulose, and carbohydrates. At 2987 cm^−1^ and 2874 cm^−1^, the S3 sample shows the antisymmetric and symmetric stretching vibration peaks of methylene and methyl C-H in lipids, respectively. The 2510 cm^−1^ peak corresponds to the C≡N stretching vibration peak in unsaturated lipids. Meanwhile, the S1 and S3 samples show an obvious narrow peak at 1799 cm^−1^, which corresponds to the C=O stretching vibration peaks of proteins, amino acids, and polypeptides. S2, S4, and S5 do not show the C=O characteristic peaks of protein and polypeptide substances near 1799 cm^−1^, which may be due to the C=O in the soil chelating with PTEs, leading to the disappearance of this peak. Samples S1–S5 exhibit different-intensity peaks at 1632 cm^−1^ and 1432 cm^−1^, which correspond to the C=O stretching vibration peak of the amide I band and the CO_3_^2−^ characteristic peak of carbonate in the soil, respectively. The intensity of the peak at 1432 cm^−1^ is weak, which further indicates that the PTEs in the soil samples have a greater impact on the total carbohydrates in the soil. In the S3 sample, the C=O stretching vibration peak of the amide I band at 1632 cm^−1^ disappears, indicating that other PTE ions were combined with the C=O stretching vibration peak, resulting in a weakening of the peak’s intensity. The peaks of the five samples at 1022 cm^−1^ are C-O stretching vibration peaks. In samples S1 and S3, the peak at 877 cm^−1^ is the characteristic peak of CrO_4_^2−^, indicating that the pollution of Cr in S1 and S3 is more serious than that in other samples. The characteristic peak of ZrO_4_^2−^ appears near 799 cm^−1^ in S1–S5. Zr is widely distributed in nature. Zirconia (ZrO_2_) is a naturally occurring raw material. Among the five soil samples, the characteristic peak intensity of ZrO_4_^2−^ is the weakest. The characteristic peak of ZnO also appears at 467 cm^−1^, indicating that there was a certain amount of ZnO pollution in the five soil samples.

#### 3.5.2. XPS Analysis

**Figure 6:**
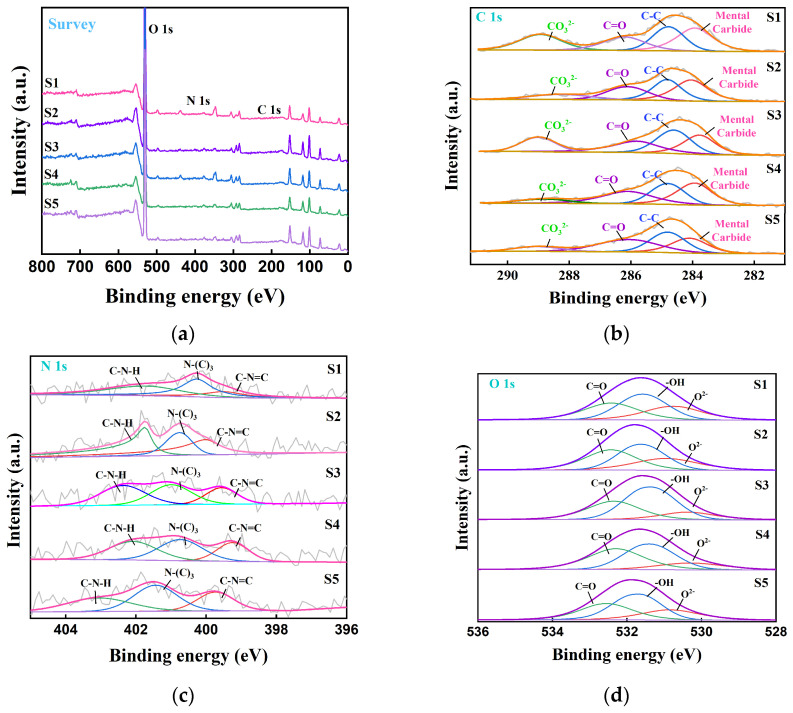
pXPS diagram of soil at five sample sites. (**a**) XPS full spectrum of five samples; (**b**) high-resolution spectrogram of C 1s; (**c**) high-resolution spectrogram of N 1s; (**d**) high-resolution spectrogram of O 1s.

To further analyze the changes in the valence states and bond energies of the elements in the PTE-contaminated areas of the site, high-resolution XPS was applied to the samples taken from five different locations. The five groups of samples contained O, N, C, and other elements, and the positions of the diffraction peaks were consistent, indicating that the elements in each area were consistent and no additional new substances were generated (Figure 6). Figure 6b shows the high-resolution C 1s spectrum. The diffraction peaks at 283.84 eV, 284.80 eV, 286.23 eV, and 288.37 eV are attributed to metal carbides, C-C, C=O, and the CO_3_^2−^ radical, respectively. It is speculated that the generation of metal carbides may have been caused by Pb and Cd pollution, and the binding energy at different positions did not shift, indicating that the chemical state of each area was consistent. Figure 6c shows the high-resolution spectrum of N 1s. The peaks at 398.75 eV, 399.76 eV, and 401.49 eV come from C-N=C, N-(C)3, and C-N-H, respectively. The binding energy and peak value at different positions show little change, consistent with element C. Figure 6d shows the high-resolution O 1s spectrum. The peaks at 530.6 eV, 531.56 eV, and 532.46 eV are mainly attributed to O^2−^, -OH, and C=O, and it is speculated that the generation of O^2−^ is mainly from the metal oxides produced by the polluted metal elements.

#### 3.5.3. XRD Analysis

**Figure 7:**
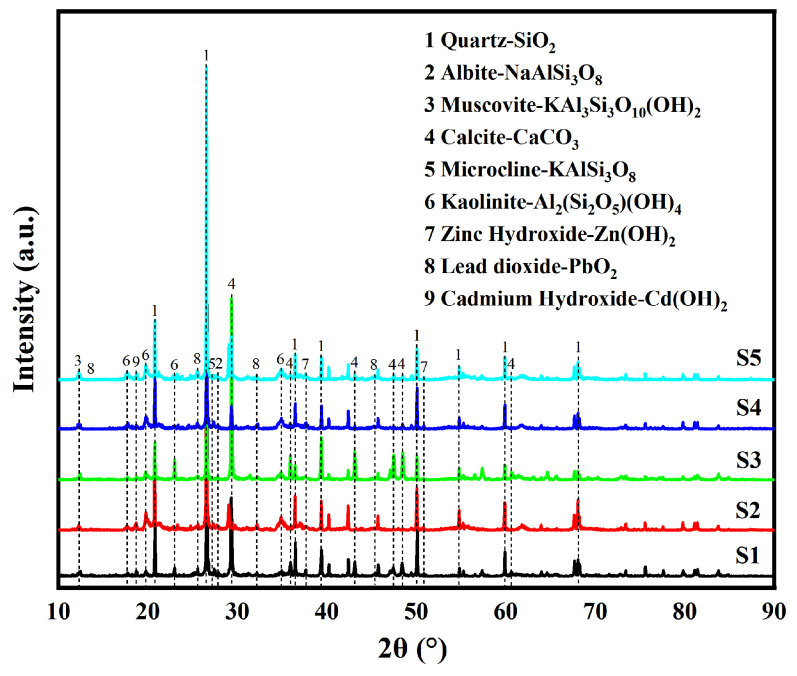
XRD diagram of soil at five sample sites.

The XRD patterns of the PTE-contaminated areas at the analysis site are shown in Figure 7. Within the diffraction ranges of the five samples, the distributions of the XRD characteristic peaks are relatively similar. The crystal peaks at the diffraction angles (2θ) of about 12.5°, 21°, and 45° are mostly consistent with the crystal plane spacing, crystal peak diffraction angle, and diffraction intensity of the Powder Diffraction File (PDF) standard card (PDF#01-079-6476). The phase indicated by the PDF standard card is kaolinite, and the corresponding crystal plane indices are (001), (-1-11), and (-203). The XRD pattern indicates kaolinite. Similarly, the weak diffraction peaks at the diffraction angle (2θ) of approximately 13.5° and 27.5° correspond to the phase potassium feldspar (PDF#01-073-9850), and the corresponding crystal plane index is (001) and (002). A small amount of quartz (PDF#01-086-2237) can be seen from the crystal peaks, with (100), (101), and (110) corresponding to the diffraction angles (2θ) of approximately 21°, 26.5°, and 36.5°. At the diffraction angles (2θ) of approximately 29°, 47.5°, and 48°, there were weak diffraction peaks. The crystal plane spacing, the crystal peak diffraction angle and diffraction intensity were consistent with those of calcite (PDF#01-072-4582), and the corresponding crystal plane indices were (104), (018), and (150). The crystal peaks at the diffraction angle (2θ) of approximately 19° and 35° are similar with the PDF standard card (PDF#01-073-0969) database in terms of crystal plane spacing, crystal peak diffraction angle, and diffraction intensity. It was determined that the XRD pattern contained cadmium hydroxide with corresponding crystal plane indices of (001) and (011), and the diffraction peak intensity was clearly apparent in samples S2, S3, and S5. At the same time, weak diffraction peaks appeared at the diffraction angles (2θ) of approximately 26°, 32°, 50°, 38°, and 51°, respectively. The corresponding substances were lead dioxide (PDF#04-002-2595), with (110), (101), and (211) facet indices, and zinc hydroxide (PDF#04-015-2996), with (011) and (102) facet indices, which appeared in the five samples. XRD pattern analysis showed that the soil in the PTE-contaminated area of the site contained quartz, kaolinite, muscovite, plagioclase, potassium feldspar, calcite, and other major components. In addition, cadmium hydroxide, lead oxide, and zinc hydroxide were found in the soil samples, indicating that the five sample sites contained multiple PTEs, including cadmium, lead, and zinc.

### 3.6. Microbial Community Analysis

#### 3.6.1. OTU Cluster Analysis

**Figure 8:**
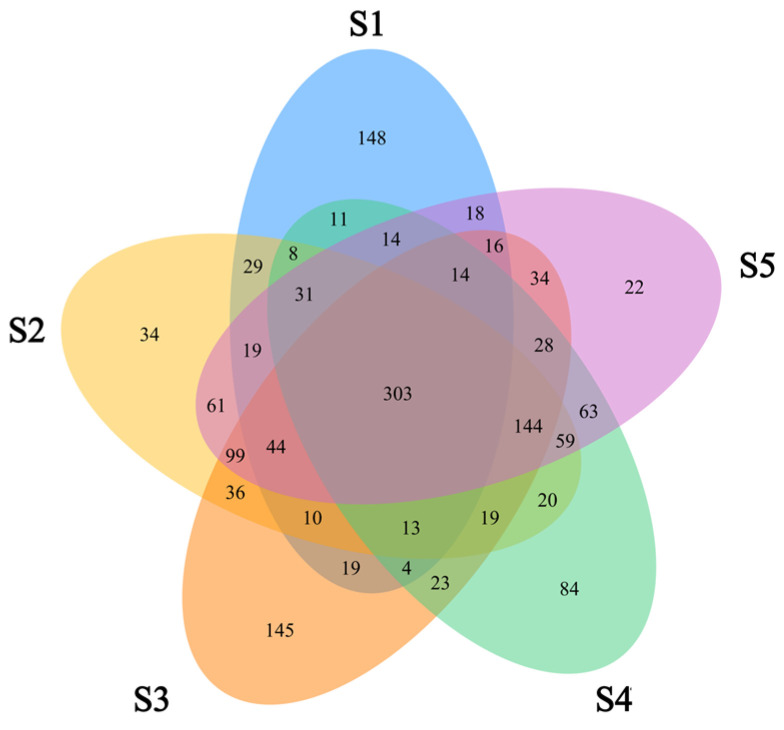
OTU clustering Venn diagram.

Significant differences were observed in microbial community structure. At the 97% nucleic acid similarity level, the five groups of samples obtained 701, 929, 951, 838, and 969 OTUs (Figure 8). The total number of OTUs in the five groups of samples was 303. The ranking of the OTU numbers is as follows: point 5 > point 3 > point 2 > point 4 > point 1. Among them, the point 5 sample contained the most OTUs, whereas points 4 and 1 contained the least. From the total number of OTUs in the samples at the four sites, the total number of OTUs at points 1234, 1235, 2345, 1245, and 2458 were 316, 347, 447, and 334, respectively, indicating that the microbial species at the four sites were the most similar.

#### 3.6.2. Diversity Index Analysis

The Shannon index of microorganisms is an index used to measure population diversity, which evaluates the diversity of microbial communities through specific calculation formulas. The Chao index is an index used to estimate the number of OTUs contained in a sample using the Chao1 algorithm. Chao1 is commonly used in ecology to estimate the total number of species. Abundance-based Coverage Estimator (ACE): The ACE index is an index used to estimate the number of operational taxonomic units (OTUs) present in a community and is one of the commonly used indices in ecology for estimating the total number of species. The ACE index is similar to the Chao1 index, both based on abundance coverage estimation methods, but their algorithms are different. The ACE index takes into account the influence of low-abundance groups. The Simpson index was proposed by Edward Hugh Simpson in 1949 and is commonly used in ecology to quantitatively describe the biodiversity of a region. The Shannon index is an indicator used to evaluate the evenness of species distribution in ecosystems. It is commonly used in conjunction with the Shannon–Wiener diversity index to comprehensively describe the biodiversity of an ecosystem [30]. Coverage refers to the microbial coverage rate, and the higher its value, the lower the probability of new species not being detected in the sample. The index actually reflects whether the sequencing results represent the true situation of the sample.

The alpha diversity index was calculated at a 97% similarity level, and results are summarized in Table 9. The Chao1 and Shannon indices reflect the richness and diversity of the microbial community. The larger the value, the higher the richness and diversity of the microbial community. The Simpson index reflects the uniformity of the microbial community. The smaller the value, the more uniform the community distribution. The Chao1 and Shannon indices of samples S1 and S5 were relatively large (Table 9), indicating that the richness and diversity of the microbial communities were high at these points, whereas the Simpson index of sample S1 was the lowest. No plant or leaching residue sites were established at site S1. The degree of PTE pollution was low, and the microbial community uniformity of the sample at this point was higher than that of the other samples with a high degree of pollution. The intake of PTE pollutants reduced soil microbial richness, resulting in low diversity and an uneven community distribution.

#### 3.6.3. Microbial Community Structure at the Phylum Level in the Samples Collected at Different Sites

**Figure 9:**
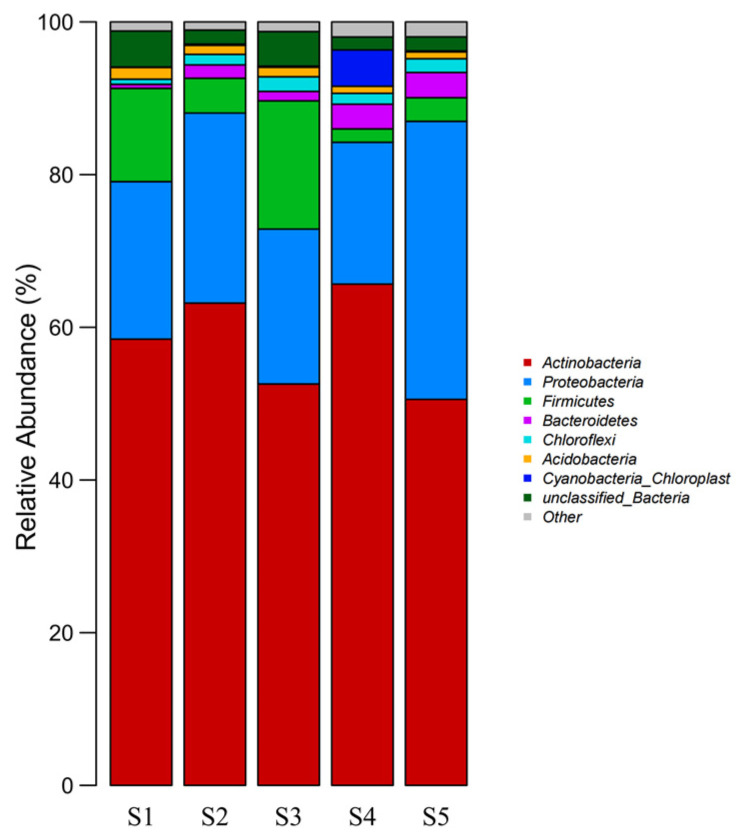
Microbial community structure at the phylum level in the samples collected at different sites.

The sequencing results were annotated to obtain information on the different sequences at different classification levels (phylum, class, order, family, and genus). Based on the annotation results, the taxon results for phyla and genera were selected for statistical analysis. The relative abundances of the dominant bacterial phyla in the soil samples from each sampling point were analyzed. *Actinobacteria* and *Proteobacteria* had the highest bacterial content at the phylum level, followed by *Firmicutes*, *Bacteroidetes*, *Chloroflexi*, *Acidobacteria*, and *Cyanobacteria_chloroplast* (Figure 9). These seven phyla accounted for more than 80% of the average total bacterial count. *Actinobacteria* had the highest relative abundance, indicating its strong adaptability to PTE-contaminated soil environments (Figure 8). *Proteobacteria* also showed an abundance advantage in the five samples. In the S1–S5 samples, the S4 samples were significantly different from the other samples at the phylum level, and the microbial compositions of the other samples were slightly different from each other. Compared with the S1 sample without plant or leaching residue sites and with low pollution, the relative abundances of *Actinobacteria* and *Proteobacteria* in S2, with a high PTE content, increased by 4.72% and 4.25%, respectively. The relative abundance of *Firmicutes* decreased by 7.65% due to an increase in the concentration of PTEs. The results showed that *Actinobacteria* and *Proteobacteria* increased and *Firmicutes* decreased in PTE-contaminated soil.

#### 3.6.4. Microbial Community Structure at the Genus Level in the Samples Collected at Different Sites

**Figure 10:**
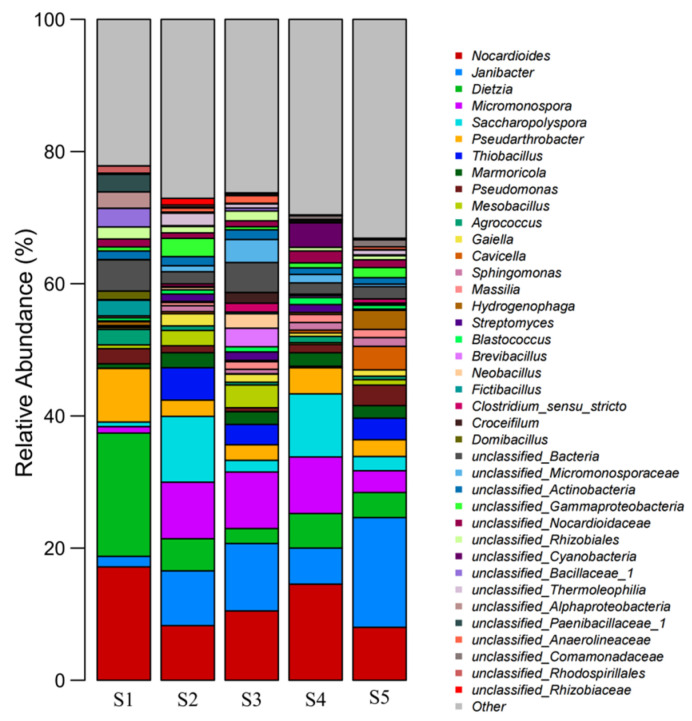
Microbial community structure at the genus level in the samples collected at different sites.

The relative abundances of the top 40 dominant bacterial species in the samples were analyzed. *Nocardioides*, *Janibacter*, *Dietzia*, *Micromonospora*, *Saccharopolypora*, and *Pseudoarthrobacter* were the dominant species (Figure 10). The abundances of these five groups of bacteria in the five samples (S1–S5) were 47.16%, 42.36%, 35.6%, 47.27%, and 36.37%, respectively. Pan et al. found that *Nocardioides* had a strong tolerance to one or more PTEs and could be used as a biological indicator of PTE pollution [31]. From S1 to S5, the proportions of *Nocardioides* were 17.15, 8.26, 10.49, 14.55, and 8.01%, respectively. The results showed that actinomycetes may be enriched in soils with a high PTE content. With an increase in metal content, the abundance of *Janibacter* groups also increased, similar to the results of the present study [32]. The content of *Janibacter* in the S1 sample without plant or leaching residue sites and with low pollution was 1.6%, and that in S2–S5 was 8.29%, 10.19%, 5.46%, and 16.60%, respectively. As the dominant species, *Dietzia*, *Micromonospora*, *Saccharopolypora*, and *Pseudoarthrobacter* all had the effect of removing high concentrations of PTEs [33,34,35].

## 4. Conclusions

(1) The average contents of Cd, Co, As, Pb, Zn, and Cu in the soil at the site were higher than the background levels in the soils of Guangxi Province. The CV of Cd, As, Pb, and Cu exceeded 1, with a high degree of dispersion, which was greatly affected by external factors.
(2) The single factor pollution index showed that Cd and As were heavily polluting, Co was slightly polluting, and the other PTEs were non-polluting. The geoaccumulation index showed that Cd was highly accumulated; Pb, As, and Co were moderately accumulated; Cu and Zn were mildly accumulated; and Ni and Cr were not accumulated. The load of Co in principal component 2 was relatively high, and there was a correlation with most other PTEs, which may be due to pollution caused by the inclusion of some of the production waste residue and exogenous soil in backfill of the site.
(3) The results of infrared spectroscopy showed that C=O in the soil was chelated with PTEs, leading to the disappearance of C=O’s characteristic peaks in protein and polypeptide substances. The PTEs in the soil samples exerted greater damage to the total carbohydrates in the soil, and there was a variable amount of PTE pollution. XPS analysis showed that metal carbides appeared in the high-resolution C 1s spectrum, O^2−^ appeared in the high-resolution O 1s spectrum, mainly from the metal oxides produced by the PTEs, and the soil was polluted with Pb, Zn, and Cd to a certain extent. The XRD results showed that cadmium hydroxide, lead oxide, and zinc hydroxide were present in all five sampling sites (S1–S5), indicating that the five samples were contaminated by PTEs such as cadmium, lead, and zinc.
(4) The risks of Cd, Zn, Cu, and Pb in the soil of the study area were high, mainly due to the joint effects of human activities and natural conditions. Measures should be taken to improve the quality of soil and the surrounding environment and reduce pollution by PTEs.
(5) *Actinobacteria* and *Proteobacteria* were the dominant microbial phyla in soil in the study area. The dominant bacterial genera were *Nocardioides*, *Janibacter*, *Dietzia, Micromonospora*, Saccharopolyspora, and Pseudarthrobacter.
(6) According to the PMF model, the soil properties in the study area are jointly influenced by agricultural activity sources, transportation and industry, and the metal smelting and electronic manufacturing industries.

## Figures and Tables

**Table 1:** Classification of single factor pollution index and Nemerow comprehensive pollution index.

Single Factor Pollution Index	Level	Nemerow Comprehensive Pollution Index	Level
$P_{i}$ ≤ 1	Class I, without pollution	$P_{Comprehensive}$ ≤ 0.7	Class I, no pollution
$P_{i}$ ∈ (1, 2]	Class II, slight pollution	$P_{Comprehensive}$ ∈ (0.7, 1.0]	Class II, slight pollution
$P_{i}$ ∈ (2, 3]	Class III, mild pollution	$P_{Comprehensive}$ ∈ (1.0, 2.0]	Class III, mild pollution
$P_{i}$ ∈ (3, 5]	Class IV, moderate pollution	$P_{Comprehensive}$ ∈ (2.0, 3.0]	Class IV, moderate pollution
$P_{i}$ > 5	Class V, severe pollution	$P_{Comprehensive}$ > 3.0	Class V, severe pollution

**Table 2:** Classification of the index of geoaccumulation (I*geo*).

I*geo*	$I_{geo}$ ≤ 0	$0\;\leq \;I_{geo}$ < 1	1 $\leq \;I_{geo}$ < 2	2 $\leq \;I_{geo}$ < 3	3 $\leq \;I_{geo}$ < 4	4 $\leq \;I_{geo}$ < 5	5 $\leq \;I_{geo}$ < 10
Level	Without accumulation	Mild to moderate accumulation	Moderate accumulation	Medium to strong accumulation	Strong accumulation	Strong to extremely severe accumulation	Extremely severe accumulation

**Table 3:** Parameter settings for PMF model construction.

Species			Category	
Co			Weak	
Cu			Weak	
Zn			Weak	
Pb			Weak	
Ni			Weak	
Cr			Weak	
As			Weak	
Cd			Weak	
Number of base runs:			20	
Base user-selected seed:			48	
Number of factors:			3	
Extra modeling uncertainty (%):			20	
	Factor 1	Factor 2	Factor 3	Unmapped
Boot Factor 1	200	0	0	0
Boot Factor 2	1	199	0	0
Boot Factor 3	8	14	178	0

**Table 4:** Characteristics of PTE content at the site.

PTE	Minimum (mg/kg)	Maximum (mg/kg)	Average (mg/kg)	Standard Deviation (mg/kg)	Coefficient of Variation	Screening Value (mg/kg)	Background Concentration
Cd	5.6	432.7	143.1	147.9	1.03	20.0	0.27
Co	12.4	93.3	47.6	31.6	0.66	20.0	10.40
As	21.5	425.2	110.2	157.2	1.42	20.0	20.50
Cr	80.6	92.5	82.8	6.5	0.07	90.0	82.10
Ni	32.5	38.6	30.3	4.4	0.14	150.0	26.60
Pb	5.1	728.8	264.3	274.2	1.04	400.0	24.00
Zn	130.9	318.6	187.9	72.6	0.39	500.0	75.60
Cu	32.5	286.9	83.5	104.0	1.24	2000.0	27.80

**Table 5:** Single factor and comprehensive pollution index table of PTEs.

PTE	Single Factor Pollution Index Range	Average Value of Single Factor Pollution Index	Pollution Index	Comprehensive Pollution Index	Pollution Level
Cd	0.279~21.635	7.154	Severe pollution	10.42	Severe pollution
Co	0.618~4.484	2.378	Slightly pollution		
As	0.074~21.261	5.312	Severe pollution		
Cr	0.228~0.805	0.625	Without pollution		
Ni	0.083~0.257	0.166	Without pollution		
Pb	0.013~1.820	0.660	Without pollution		
Zn	0.261~0.637	0.375	Without pollution		
Cu	0.001~0.143	0.038	Without pollution		

**Table 6:** Site’s soil PTE indices of geoaccumulation (I*geo*).

PTE	Index of Geoaccumulation	Pollution Level
Cu	0.895	Mild to moderate accumulation
Zn	0.729	Mild to moderate accumulation
Pb	2.876	Medium to strong accumulation
Ni	−0.675	Without accumulation
Cr	−1.526	Without accumulation
As	1.789	Moderate accumulation
Co	1.608	Moderate accumulation
Cd	8.481	Extremely severe accumulation

**Table 7:** Correlation table of PTEs in soil.

Pearson	Cu	Zn	Pb	Ni	Cr	As	Co	Cd
Cu	1.000							
Zn	−0.322	1.000						
Pb	0.776	0.347	1.000					
Ni	0.776	0.347	1.00 **	1.000				
Cr	0.405	0.726	0.884 *	0.884 *	1.000			
As	0.681	0.473	0.990 **	0.990 *	0.942 *	1.000		
Co	0.643	0.516	0.981 **	0.981 *	0.959 *	0.999 **	1.000	
Cd	0.587	0.574	0.964 **	0.964 *	0.977 **	0.992 **	0.997 **	1.000

Note: ** indicates extremely significant correlation (*p* < 0.01), * indicates significant correlation (0.01 < *p* < 0.05), and *p* > 0.05 indicates no correlation.

**Table 8:** Soil PTE principal component analysis.

PTE	Component 1	Component 2	Component 3
Zn	−0.402	0.124	−0.860
Pb	−0.841	−0.005	0.464
Cr	−0.245	0.360	0.842
Co	0.632	0.739	0.181
Cd	0.570	−0.811	0.103
Cu	0.767	0.400	−0.081
Ni	0.899	0.172	0.054
As	0.374	−0.890	0.249

**Table 9:** APLHA diversity index.

Sample Number	Chao1	Shannon	ACE	Simpson	Shannoneven	Coverage
1	746.69	4.26	750.33	0.05	0.65	1.00
2	998.07	4.62	986.71	0.03	0.78	1.00
3	965.35	4.81	965.30	0.03	0.70	1.00
4	872.17	4.66	861.87	0.03	0.69	1.00
5	1094.20	4.72	1088.85	0.04	0.69	1.00

## Data Availability

The original contributions presented in this study are included in the article, and further inquiries can be directed to the corresponding authors.
